# Combined Healthy Lifestyle Is Inversely Associated with Psychological Disorders among Adults

**DOI:** 10.1371/journal.pone.0146888

**Published:** 2016-01-15

**Authors:** Parvane Saneei, Ahmad Esmaillzadeh, Ammar Hassanzadeh Keshteli, Hamid Reza Roohafza, Hamid Afshar, Awat Feizi, Peyman Adibi

**Affiliations:** 1 Food Security Research Center, Isfahan University of Medical Sciences, Isfahan, Iran; 2 Students’ Research Committee, Isfahan University of Medical Sciences, Isfahan, Iran; 3 Department of Community Nutrition, School of Nutrition and Food Science, Isfahan University of Medical Sciences, Isfahan, Iran; 4 Department of Community Nutrition, School of Nutritional Sciences and Dietetics, Tehran University of Medical Sciences, Tehran, Iran; 5 Department of Medicine, University of Alberta, Edmonton, Alberta, Canada; 6 Integrative Functional Gastroenterology Research Center, Isfahan University of Medical Sciences, Isfahan, Iran; 7 Psychosomatic Research Center, Department of Psychiatry, Isfahan University of Medical Sciences, Isfahan, Iran; 8 Department of Epidemiology and Biostatistics, School of Public Health, Isfahan University of Medical Sciences, Isfahan, Iran; National Cancer Center, JAPAN

## Abstract

**Background and Aims:**

Joint association of lifestyle-related factors and mental health has been less studied in earlier studies, especially in Middle Eastern countries. This study aimed to examine how combinations of several lifestyle-related factors related to depression and anxiety in a large group of middle-age Iranian population.

**Methods:**

In a cross-sectional study on 3363 Iranian adults, a healthy lifestyle score was constructed by the use of data from dietary intakes, physical activity, smoking status, psychological distress and obesity. A dish-based 106-item semi-quantitative validated food frequency questionnaire (FFQ), General Practice Physical Activity Questionnaire (GPPAQ), General Health Questionnaire (GHQ) and other pre-tested questionnaires were used to assess the components of healthy lifestyle score. The Hospital Anxiety and Depression Scale (HADS) was applied to screen for anxiety and depression.

**Results:**

After adjustment for potential confounders, we found that individuals with the highest score of healthy lifestyle were 95% less likely to be anxious (OR: 0.05; 95% CI: 0.01–0.27) and 96% less likely to be depressed (OR: 0.04; 95% CI: 0.01–0.15), compared with those with the lowest score. In addition, non-smokers had lower odds of anxiety (OR: 0.64; 95% CI: 0.47–0.88) and depression (OR: 0.62; 95% CI: 0.48–0.81) compared with smokers. Individuals with low levels of psychological distress had expectedly lower odds of anxiety (OR: 0.13; 95% CI: 0.10–0.16) and depression (OR: 0.10; 95% CI: 0.08–0.12) than those with high levels. Individuals with a healthy diet had 29% lower odds of depression (OR: 0.71; 95% CI: 0.59–0.87) than those with a non-healthy diet.

**Conclusion:**

We found evidence indicating that healthy lifestyle score was associated with lower odds of anxiety and depression in this group of Iranian adults. Healthy diet, psychological distress, and smoking status were independent predictors of mental disorders.

## Introduction

The common psychological disorders, depression and anxiety, are major public health problems around the world [1]. Approximately, 20% of U.S. adults report depressive symptoms and the prevalence is increasing drastically [2]. The increased rates of depression may be due, in part, to improvements in diagnostic recognition, increased community acceptance of this condition [2] and contemporary lifestyles [3]. Depression contributes to many disabilities, chronic disorders [4–6] and mortality [7]. It could adversely affect health status, quality of life and ability to work [8, 9]. The prevalence of anxiety among Iranian adults is approximately 20.8% [10]. Anxiety disorder is often accompanied by general somatic symptoms such as fatigue, loss of energy, feeling slowed up or agitated impaired physical, restless and role functioning [11].

The precise etiology of depression or anxiety is not known; however, environmental, psychological, and genetic factors might contribute to these conditions [12]. Several modifiable risk factors, including obesity, physical inactivity, smoking and poor diet, have been studied in association with these disorders; however, results were conflicting [6, 13–17]. For example, a cross-sectional study on a nationally-representative sample of U.S. population has identified lower odds of depression in physically active overweight/obese adults, but not in normal-weight individuals [14].

Several studies have examined modifiable lifestyle-related factors with mental illnesses; most have focused on the relation of a certain behavior, rather than combined lifestyle factors, and depression or anxiety. Joint association of lifestyle-related factors, or the combined healthy lifestyle score, and mental health has been less studied in earlier studies. In an investigation among a subgroup of the National Health and Nutrition Examination Survey (NHANES) population [18], the associations between dietary intakes, physical activity, and smoking status, as independent predictors, with depression were examined. They found a dose–response relationship between concurrent occurrence of these behaviors and depressive symptoms [18]; however, obesity and psychological distress, as two major risk factors for mental disorders, were not taken into account in that study. Studying the association between lifestyle-related factors and mental health is particularly relevant for the understudied region of the Middle East, where these factors are different from other parts of the world. Due to cultural expectations women have significantly lower physical activity levels and discretionary exercise than men in this region [19]. The rate of smoking among men in developing countries is similar to western nations, while this is less prevalent in women (22% in men vs. 2.1% in women in Iran) [20]. In terms of dietary intakes, more than 55% of energy intake in Iranian population is derived from carbohydrates, especially refined grains [21]. Furthermore, prior studies assessing lifestyle-related factors and depression or anxiety have mostly focused on elderly populations [13, 22] or patients with chronic diseases [4, 7, 23, 24] and the beneficial effects of adherence to multiple components of a healthy lifestyle in apparently healthy middle-age individuals have not been systematically examined. The aim of the current study was to examine how combinations of several lifestyle-related factors relate to depression and anxiety in a large group of middle-age Iranian adults.

## Materials and Methods

### Participants

This cross-sectional study was done within the framework of the Study on the Epidemiology of Psychological-Alimentary Health and Nutrition (SEPAHAN) [25], a project that was performed on Iranian general adults working in 50 health centers affiliated to Isfahan University of Medical Sciences (IUMS). In order to increase the participation rate, the accuracy of collected data and to decrease participants’ fatigue, the project included two main phases. In the first phase, a detailed self-administered questionnaire on socio-demographic factors and dietary behaviors was distributed among 10087 apparently healthy adults and 8691 individuals returned the completed questionnaires (response rate: 86.16%). No significant difference was found between the demographic data of those who returned the completed questionnaires and those who did not. In the second phase, information on psychological distress and mental disorders was collected using validated questionnaires (response rate: 64.64%). After merging data from these two phases, we had complete information for 3863 participants. Data for 2376 participants could not be used in merging process, because: 1) some participants had no information at phase one (did not complete the questionnaires at first phase), 2) some did not complete their identification code in phase 1 or 2 which prohibited us merging their data; 3) some had missing data on exposure, outcome or covariate variables. In addition, in the current study, participants with caloric intakes outside the range of 800–4200 kcal/day were excluded (n = 500). These exclusions resulted in a dataset of 3363 adults who had complete information on lifestyle behaviors and mental health. All participants provided signed informed written consent. The study was ethically approved by the Medical Research Ethics Committee of Isfahan University of Medical Sciences (IUMS), Isfahan, Iran.

### Assessment of exposure

Data on dietary intakes were collected using a Willett-format dish-based 106-item semi-quantitative food frequency questionnaire, which was designed and validated specifically for Iranian adults [26]. Detailed information about the design, foods included as well as the validity of this questionnaire has been reported elsewhere [26]. Briefly, the FFQ contained information on frequency of consumption of foods or dishes over the last year, along with common portion sizes used in Iran. A daily value for each item was calculated based on food composition, specified portion size and the average of reported frequency. Nutrient intakes were calculated by summing up the nutrient contents of all foods and dishes. Nutritionist IV software, that was modified for Iranian foods, was used to obtain nutrient intakes of each participant. Overall, our previous investigations indicated that the FFQ provides reasonably valid and reliable measures of the average long-term intakes of foods [27], food groups [28] and nutrients [29].

Data on weight (in kilograms) and height (in centimeters) were obtained using a self-reported questionnaire. Body mass index was calculated as weight in kilograms divided by the square of height in meters. In our validation study on 200 participants from the same population, we found that the correlation coefficient between self-reported and technician-measured weight and height were 0.95 (P<0.001) and 0.83 (P<0.001), respectively. The correlation coefficient for computed BMI from self-reported values and the one from measured values was 0.70 (P<0.001). These findings indicate that the self-reported values of anthropometric indicators provide reasonably valid measures in this population. Participants were classified into two categories based on their BMI: normal weight (18.5–24.9 kg/m^2^), overweight or obese (≥ 25.0 kg/m^2^).

Physical activity of study participants were assessed by the use of a General Practice Physical Activity Questionnaire (GPPAQ). This questionnaire is a simple validated screening tool for ranking adult people’s physical activity with focusing on current general activities [30]. Participants were classified into 4 categories: active (>3 h/week), moderately active (1–3 h/week), moderately inactive (<1 h/week), and inactive (no physical activity), based on the type and intensity of their physical activity in work hours and during the weekends. The validity of the GPPAQ for assessment of habitual physical activity levels has earlier been shown [30].

The Iranian validated version of General Health Questionnaire (GHQ) with 12-items was used to assess psychological distress [31]. GHQ-12 is a brief, simple, easy-to-complete instrument for measuring current and primary mental health that asks the respondents whether they have experienced a particular symptom of psychological distress recently. Each item consists of a four-point scale (less than usual, no more than usual, rather more than usual, or much more than usual). There are two most common scoring methods, bimodal (0-0-1-1) and Likert scoring (0-1-2-3) and it gives a total score of 12 or 36 on the basis of the scoring method selected. We used the bimodal scoring style for this study. This gives scores ranging from 0 to 12. Higher scores indicate a greater degree of psychological distress. In the current study, high levels of psychological distress were defined as having the score of 4 or more [31]. Information on cigarette smoking was assessed by a pre-tested questionnaire.

### Classification of low risk categories

To construct a healthy lifestyle score, data from dietary intakes, physical activity, smoking status, psychological distress and obesity were used. With regards to healthy diet, we used the previously designed Alternative Healthy Eating Index-2010 (AHEI-2010) [32]. The index was composed of 11 components [fruit, vegetables, whole grains, nuts and legumes, long chain omega-3 fats (docosahexaenoic acid and eicosapentaenoic acid), polyunsaturated fatty acids, alcohol consumption, sugar sweetened drinks and fruit juice, red and processed meats, trans fat, and sodium] [32]. In the current study, alcohol consumption was not included into the score, due to lack of information in the original dataset. To construct the index, first we obtained energy-adjusted intakes of the mentioned components based on residual method [33]. Then, participants were classified based on the deciles categories of energy-adjusted intakes of these components. Since scoring by deciles would be least prone to misclassification, we used deciles categories of components instead of quantitative classifications. Individuals in the highest deciles of fruits, vegetables, whole grains, nuts and legumes, long chain omega-3 fats and polyunsaturated fatty acids were given the score of 10 and those in the lowest deciles of these items were given the score of 1. Individuals in the other deciles of these components were assigned the corresponding scores. Regarding sugar sweetened drinks and fruit juice, red and processed meat, trans fat, and sodium intake, the lowest deciles were given a score of 10 and the highest deciles were given the score of 1. Those in deciles 9, 8, 7, 6, 5, 4, 3 and 2 of these components were given the scores of 2, 3, 4, 5, 6, 7, 8 and 9, respectively. To compute the AHEI-2010, we added the scores for the individual items, resulting in a minimum score of 10 and a maximum score of 100. Participants in the highest 40% of AHEI-2010 (upper two fifths) were considered to have a healthy diet.

For physical activity, we defined low risk group as individuals with active and moderately active lifestyle. With regard to cigarette smoking, ex-smokers and individuals who never smoked were defined as low risk group. Participants with a GHQ score of 3 or less were defined as a low risk group in terms of psychological distress. In terms of BMI status, those with a BMI< 25 kg/m^2^ were considered as low risk group.

**Fig 1:**
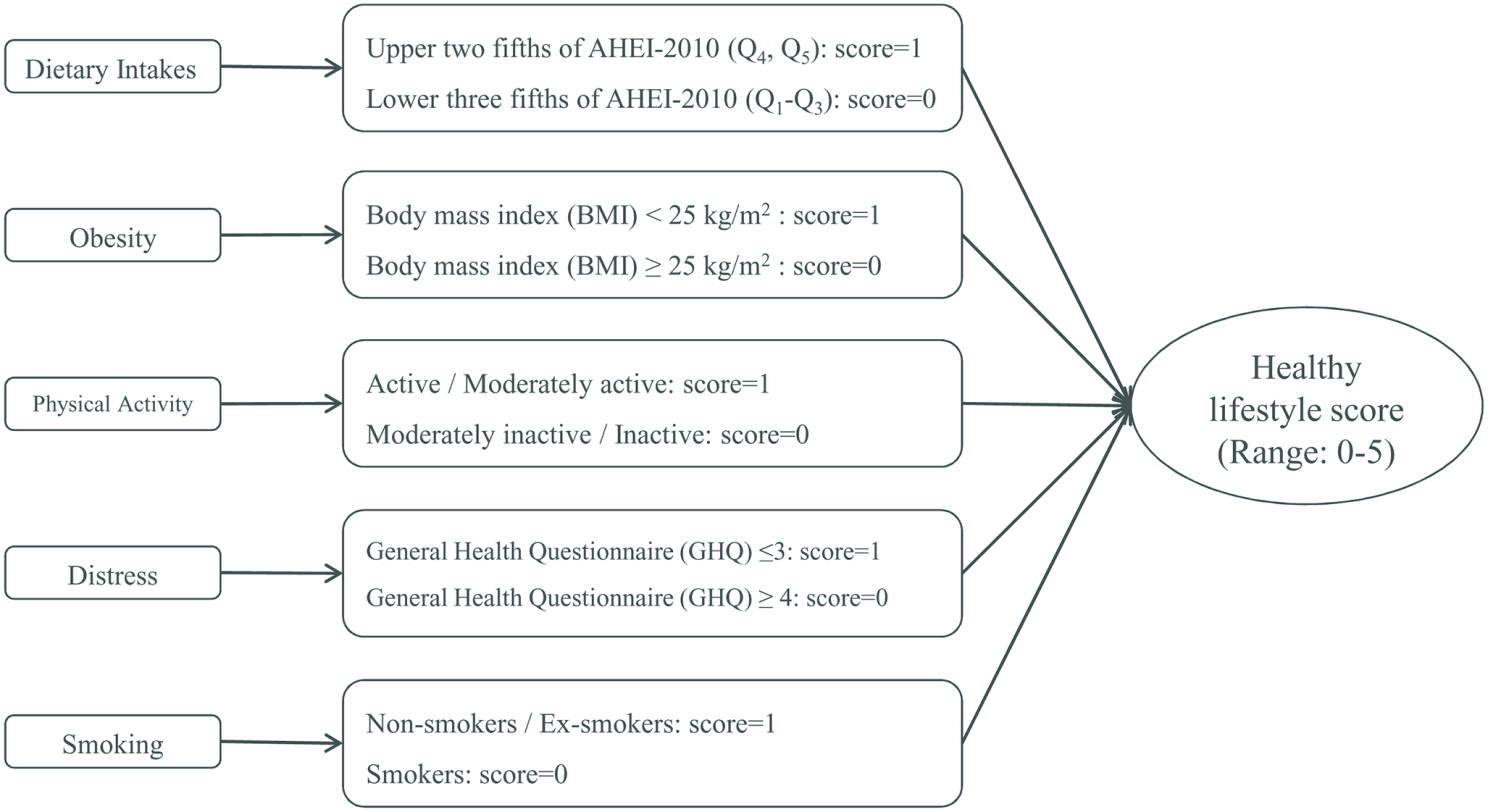
The healthy lifestyle score development.

Fig 1 shows how the healthy lifestyle score was developed. This score was constructed through summing up the scores that each participant obtained for components of lifestyle as mentioned above, given that subjects in the low risk categories of the mentioned components received the score of 1 and others received the score of 0. Therefore, a composite global healthy lifestyle score ranged from 0 to 5.

### Assessment of outcome

The Iranian validated version of Hospital Anxiety and Depression Scale (HADS) was used to screen for anxiety and depression [34]. HADS is a brief and useful questionnaire to measure psychological disorders and assess the symptom severity of anxiety disorders and depression. The HADS contains 14 items and consists of two subscales: anxiety and depression. Each item includes a four-point scale; higher scores indicate an elevated level of anxiety and depression. Maximum score is 21 for anxiety and depression. Scores of 8 or more on either subscale were considered as psychological disorders and scores of 0–7 were defined as ''normal" in the current study [34].

### Assessment on confounders

Additional covariate information regarding age, gender, marital status, education levels, family size, house possession, disease history, current use of anti-depressants medications (including nortriptyline, amitriptyline or imipramine, fluoxetine, citalopram, fluvoxamine and sertraline) and dietary supplements (including intake of iron, calcium, vitamins and other dietary supplements) was obtained using self-administered questionnaires.

### Statistical Analysis

Subjects were categorized based on their scores of healthy life-style. To compare general characteristics and dietary intakes across different categories of lifestyle score, we used one-way ANOVA and chi-square tests where appropriate. We computed age-, gender- and energy-adjusted intakes of nutrients and food groups using analysis of covariance (ANCOVA). Comparison of dietary intakes across levels of healthy lifestyle score was done using ANCOVA with Bonferroni correction. To find the relation between healthy life-style and odds of anxiety and depression, we used multivariate logistic regression in different models. First, we controlled for age (years) and sex (male/female). Additional adjustment was done for marital status (single/married), educational level (>diploma/≤diploma), family size (>4/≤4 members), house possession (yes/no), diabetes (yes/no), current use of anti-depressants medications (yes/no) and dietary supplements (yes/no) in the second model. To calculate the trend of odds ratios across increasing scores of healthy life-style, we considered healthy life-style scores as an ordinal variable. In these analyses, those with zero score of healthy lifestyle were considered as the reference category. To determine the association of individual components of healthy lifestyle score with outcomes, we constructed crude and multivariable-adjusted models controlling for above-mentioned covariates, as well as other components of healthy life style score for each component. Two-tailed P values less than 0.05 were considered to be statistically significant. Statistical package for the social sciences (SPSS) software, version 18 was used for all analyses.

## Results

Mean age of study participants was 36.3±7.9 years, and 58.3% (n = 1960) were females. The prevalence of anxiety and depression was 15.2% (males 10.8% and females 18.3%) and 30.0% (males 22.9% and females 35.1%), respectively. General characteristics of study participants across different levels of healthy lifestyle score are presented in Table 1. Compared with those with a zero score of healthy lifestyle, individuals in the highest score were younger (36.7±9.2 vs. 37.4±6.3, P = 0.01), less likely to be married (75.8% vs. 76.9%, P<0.001), diabetic (3.0% vs. 11.1%, P = 0.01), to take anti-depressants medications (4.5% vs. 18.5%, P<0.001) and had lower weight (65.8±8.5 vs. 75.8±8.9, P<0.001) and body mass index (22.6±1.9 vs. 28.5±2.5, P<0.001).

**Table 1:** General characteristics of study participants across different levels of healthy lifestyle score ^a^.

	Healthy lifestyle scores						
	0	1	2	3	4	5	P^b^
Age (y)	37.4±6.3	37.7±7.1	36.7±7.4	36.0±8.2	35.5±7.9	36.7±9.2	0.01
Weight (kg)	75.8±8.8	75.3±13.1	71.8±14.1	67.1±12.5	63.2±10.4	65.8±8.5	<0.001
Body mass index (kg/m2)	28.5±2.5	28.2±5.6	26.0±4.2	24.5±4.5	22.9±2.6	22.6±1.9	<0.001
Female (%)	63.0	58.1	57.7	59.0	60.1	34.3	0.01
Married (%)	76.9	89.1	86.6	80.1	73.9	75.8	<0.001
Education (% > diploma)	50.0	50.9	58.4	62.9	67.1	58.5	<0.001
Family size (% >4)	14.8	13.0	11.8	13.3	12.2	17.9	0.70
House possession (%)	44.4	55.8	56.4	59.7	59.4	65.7	0.77
Diabetes (%)	11.1	1.7	2.1	1.6	1.2	3.0	0.01
Anti-depressants medications intake^c^ (%)	18.5	12.3	6.8	3.6	4.1	4.5	<0.001
Dietary supplements intake^d^ (%)	37.0	30.2	28.6	30.7	31.1	23.9	0.62
Smokers (%)	100.0	42.5	19.6	7.3	1.9	0.0	<0.001
Physically inactive (%, <1 hours/w)	100.0	99.0	95.4	89.1	69.19	0.0	<0.001
Overweight or obese^e^ (%)	100.0	89.4	64.7	36.1	10.3	0.0	<0.001
High levels of distress^f^ (%)	100.0	75.1	35.7	9.6	2.4	0.0	<0.001

^a^All values are means ± standard deviation (SD), unless indicated.

^b^Obtained from ANOVA for continuous variables and chi-square test for categorical variables

^c^Anti- depressants medications included the intake of nortriptyline, amitriptyline or imipramine, fluoxetine, citalopram, fluvoxamine and sertraline.

^d^Dietary supplements included the intake of iron, calcium, vitamins and other dietary supplements.

^e^BMI≥25

^f^GHQ score≥4

Dietary intakes of selected nutrients and food groups of study participants across different levels of healthy lifestyle score are provided in Table 2. Higher scores of healthy lifestyle were associated with higher intakes of dietary fiber, omega-3 fatty acids, vitamin B_6_, and lower intakes of low-fat dairy and refined grains. No significant difference was observed in total energy and macro-nutrient intakes of study participants across different scores of healthy lifestyle.

**Table 2:** Dietary intakes of selected nutrients and food groups of study participants across different levels of healthy lifestyle score ^a^.

	Healthy lifestyle scores						
	0	1	2	3	4	5	P^b^
Energy (Kcal/d)	2198.7±160.1	2347.9±50.1	2387.9±27.3	2381.9±23.4	2396.4±35.8	2416.2±108.4	0.84
Nutrients:							
Proteins (% of energy)	14.5±0.5	14.7±0.1	14.8±0.1	14.9±0.1	15.0±0.1	15.1±0.3	0.39
Fats (% of energy)	38.9±1.3	37.7±0.4	37.5±0.2	37.5±0.2	37.3±0.3	37.3±0.9	0.85
Carbohydrates (% of energy)	47.6±1.5	48.8±0.5	49.0±0.3	49.1±0.2	49.6±0.3	49.7±1.0	0.48
Dietary fiber (g/d)	19.7±1.0	21.0±0.3	21.7±0.2	22.8±0.2	24.3±0.2	26.0±0.7	<0.001
Omega-3 fatty acids (g/d)	1.57±0.14	1.63±0.04	1.69±0.02	1.74±0.02	1.89±0.03	1.99±0.09	<0.001
Vitamin B_1_ (mg/d)	1.83±0.11	1.87±0.03	1.91±0.02	1.84±0.02	1.77±0.03	1.69±0.07	<0.001
Vitamin B_6_ (mg/d)	1.84±0.08	1.91±0.02	1.94±0.01	1.99±0.01	2.07±0.02	2.19±0.05	<0.001
Iron (mg/d)	17.3±0.6	18.0±0.2	18.0±0.1	17.6±0.1	16.9±0.1	16.9±0.4	<0.001
Food groups:							
Red meat (g/d)	80.3±7.8	84.2±2.5	81.9±1.3	78.5±1.1	70.6±1.8	73.8±5.3	<0.001
Whole grains (g/d)	25.9±14.7	36.2±5.6	36.5±2.5	42.5±2.1	56.9±3.3	47.6±9.9	<0.001
Fruit (g/d)	188.5±39.7	217.7±12.4	232.9±6.8	286.7±5.8	366.3±8.9	415.1±26.9	<0.001
Vegetables (g/d)	200.0±22.6	214.3±7.1	224.5±3.9	242.7±3.3	263.7±5.1	310.0±15.3	<0.001
Nuts and legumes (g/d)	50.4±7.0	54.4±2.2	53.9±1.2	58.2±1.0	61.2±1.6	69.4±4.8	<0.001
Low-fat dairy (g/d)	402.4±50.4	304.0±15.8	313.7±8.6	339.8±7.3	366.1±11.6	348.7±34.1	0.001
Refined grains (g/d)	451.1±31.4	421.5±9.8	425.9±5.4	388.8±4.6	337.3±7.0	301.6±21.3	<0.001

^a^All values are means ± standard error (SE); energy intake is adjusted for age and gender, all other values are adjusted for age, gender and energy intake.

^b^Obtained from ANCOVA.

**Fig 2:**
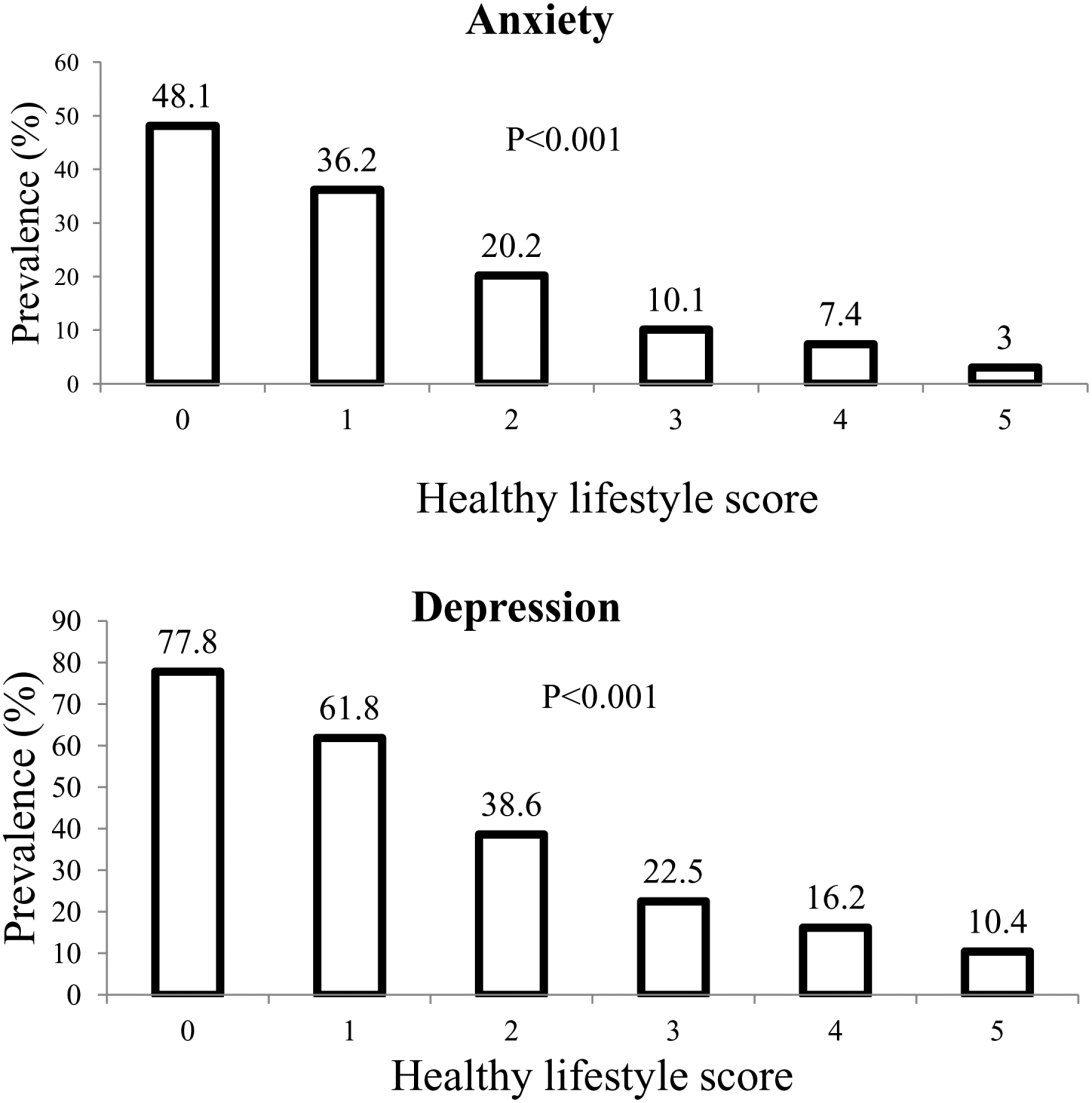
The prevalence of anxiety and depression across different levels of healthy lifestyle score. Hospital Anxiety and Depression Scale (HADS) score≥8 were considered as being anxious or depressed.

The prevalence of anxiety and depression across different levels of healthy lifestyle score is shown in Fig 2. Individuals with the greatest score of healthy lifestyle were less likely to be anxious (3.0 vs. 48.1%, P<0.001) and depressed (10.4 vs. 77.8%, P<0.001) compared with those with the lowest score.

Multivariable-adjusted odds ratios (ORs) for anxiety and depression across different levels of healthy lifestyle score are presented in Table 3. Subjects in the top category of healthy lifestyle score had lower odds for anxiety [OR: 0.03; 95% confidence interval (CI) 0.01–0.16], and depression (OR: 0.03; 95% CI: 0.01–0.11), compared with those in the bottom category. After taking potential confounding into account, we found that individuals with the highest score of healthy lifestyle were 95% less likely to be anxious (OR: 0.05: 95% CI: 0.01–0.27) and 96% less likely to be depressed (OR: 0.04: 95% CI: 0.01–0.15), compared with those with the lowest score. In addition, a decreasing trend in the odds of anxiety and depression was seen with increasing scores of healthy lifestyle.

**Table 3:** Multivariable- adjusted odds ratio for anxiety and depression across different levels of healthy lifestyle score^1^.

	Healthy lifestyle score						
	0	1	2	3	4	5	P_trend_^2^
Anxiety							
Crude	1.00	0.61 (0.28–1.35)	0.27 (0.13–0.59)	0.12 (0.06–0.26)	0.09 (0.04–0.20)	0.03 (0.01–0.16)	<0.001
Model 1	1.00	0.59 (0.26–1.35)	0.26 (0.12–0.57)	0.11 (0.05–0.25)	0.08 (0.04–0.19)	0.04 (0.01–0.21)	<0.001
Model 2	1.00	0.61 (0.25–1.49)	0.26 (0.11–0.62)	0.12 (0.05–0.29)	0.09 (0.04–0.24)	0.05 (0.01–0.27)	<0.001
Depression							
Crude	1.00	0.46 (0.18–1.18)	0.18 (0.07–0.45)	0.08 (0.03–0.21)	0.06 (0.02–0.14)	0.03 (0.01–0.11)	<0.001
Model 1	1.00	0.51 (0.20–1.34)	0.19 (0.08–0.49)	0.09 (0.03–0.22)	0.06 (0.02–0.14)	0.03 (0.01–0.12)	<0.001
Model 2	1.00	0.53 (0.18–1.51)	0.21 (0.07–0.58)	0.09 (0.03–0.26)	0.06 (0.02–0.17)	0.04 (0.01–0.15)	<0.001

^1^All values are odds ratios and 95% confidence intervals. Hospital Anxiety and Depression Scale (HADS) score≥8 were considered as being anxious or depressed. Model 1: Adjusted for age and gender. Model 2: Further adjustment for marital status, education, family size, house possession, diabetes, intake of anti-depressants medications and dietary supplements.

^2^Obtained by the use of categories of healthy lifestyle score as an ordinal variable in the model.

Multivariable-adjusted odds ratios for anxiety and depression across different levels of individual components of healthy lifestyle score are provided in Table 4. After controlling for potential confounders, non-smokers had lower odds of anxiety (OR: 0.64; 95% CI: 0.47–0.88) and depression (OR: 0.62; 95% CI: 0.48–0.81) compared with smokers. Individuals with low levels of psychological distress had expectedly lower odds of anxiety (OR: 0.13; 95% CI: 0.10–0.16) and depression (OR: 0.10; 95% CI: 0.08–0.12) than those with high levels. Individuals with a healthy diet had 29% lower odds of depression (OR: 0.71; 95% CI: 0.59–0.87) than those with a non-healthy diet. In crude model, subjects with a healthy diet had reduced chance for anxiety (OR: 0.75; 95% CI: 0.61–0.91), compared with those that had a non-healthy diet; however, this association attenuated after adjustment for potential confounders (OR: 0.82; 95% CI: 0.64–1.04). No significant associations were seen between physical activity levels or BMI status and anxiety and depression in crude or multivariable-adjusted model.

**Table 4:** Multivariable- adjusted odds ratio for anxiety and depression across different levels of components of healthy lifestyle score ^a^.

Components of healthy lifestyle score	Anxiety	Depression
Non-smokers vs. smokers^b^		
Crude	0.66 (0.52–0.85)	0.67 (0.55–0.83)
Multivariable-adjusted model	0.64 (0.47–0.88)	0.62 (0.48–0.81)
Physically active vs. physically inactive^c^		
Crude	0.99 (0.75–1.31)	0.81 (0.64–1.01)
Multivariable-adjusted model	1.37 (0.96–1.94)	0.97 (0.72–1.30)
Normal-weight vs. overweight or obese^d^		
Crude	0.86 (0.71–1.04)	0.95 (0.82–1.10)
Multivariable-adjusted model	0.89 (0.70–1.14)	0.93 (0.76–1.13)
Low levels of distress vs. high levels of distress^e^		
Crude	0.12 (0.10–0.15)	0.09 (0.08–0.11)
Multivariable-adjusted model	0.13 (0.10–0.16)	0.10 (0.08–0.12)
Healthy diet vs. non-healthy diet^f^		
Crude	0.75 (0.61–0.91)	0.71 (0.61–0.83)
Multivariable-adjusted model	0.82 (0.64–1.04)	0.71 (0.59–0.87)

^a^All values are odds ratios and 95% confidence intervals. Hospital Anxiety and Depression Scale (HADS) score≥8 were considered as being anxious or depressed. Multivariable-adjusted model: Adjustments for age and gender, marital status, education, family size, house possession, diabetes, intake of anti-depressants medications and dietary supplements, as well as other components of healthy life style score.

^b^Ex-smokers were included in the group of non-smokers

^c^Physical activity ≥1 hours/w vs. <1 hour/w

^d^BMI<25 vs. BMI≥25 kg/m^2^.

^e^GHQ score <4 vs. GHQ score ≥4.

^f^Upper two fifths vs. lower three fifths of AHEI-2010.

## Discussion

In this cross-sectional study in a large group of Iranian adults, we found a significant negative association between healthy lifestyle score and odds of anxiety and depression, even after adjustment for potential confounders. Non-smokers, individuals with low levels of psychological distress and those with a healthy diet had reduced chance for anxiety and depression, compared with smokers, subjects with high levels of psychological distress and subjects with a non-healthy diet, respectively. To the best of our knowledge, this study is the first examining the association between combined healthy lifestyle factors and mental disorders in a Middle Eastern population.

Anxiety and depression may, in part, be influenced by genetics [12]; however, there is also substantial evidence demonstrating that these disorders could be modified through environmental and psychological-based strategies [12, 14, 15, 17, 18]. Our data indicated that occurrence of multiple health-enhancing behaviors may play an important role in preventing anxiety and depression. Given the size of the burden of illnesses, the benefits of even a minimal impact on the prevalence of depression or anxiety will be substantial to the entire population. Multiple behavior changes might also be efficient in reducing mental disorders among those with diagnosed anxiety or depression.

Few previous studies have examined combinations of lifestyle factors in relation to psychological disorders. In a cross-sectional study among a national sample of U.S. adults, Loprinzi et al have indicated that the combination of healthy habits including physical activity, healthy diet, and smoking status was associated with depression symptoms [18]; with the greatest reduction in odds of depression (82%) among those with all three healthy behaviors and a substantive reduction (67%) in individuals with two healthy behaviors. Another cross-sectional study on 1612 patients at risk of cardiovascular disease reported a significant association between a cluster of unhealthy behaviors and anxiety and depression [23]; patients who presented anxious or depressive symptoms were more likely to be inactive and to have a poor diet than non-anxious or non-depressed subjects. However, there was no significant correlation between anxiety and smoking habits in women in that study [23]. Thus, our findings were consistent with those from previous publications in developed countries. However, earlier studies have not considered psychological distress as a component of the lifestyle factors. This is particularly important for high stress settings like developing countries.

In the current study, we found a reduced risk of anxiety and depression among those with a healthy diet. An emerging body of evidence has suggested that dietary patterns play important roles in mental health [35–38]. In 2013, in a population-based study of over 2000 adult Japanese employees, Suzuki et al. [37] reported that the Japanese dietary pattern was consistently protective against depressive symptoms, while other dietary patterns were not associated with depressive symptoms. In a Chinese study in young adolescents, healthy dietary habits were associated with reduced psychological symptoms, whilst unhealthy dietary habits had a positive relationship with such symptoms [38]. Subsequent investigations in an Iranian population demonstrated that greater adherence to the lacto-vegetarian dietary pattern was protectively associated with depression in women, whereas the Western dietary pattern was associated with increased odds of depression in men [35]. Such findings were also demonstrated by prospective studies [39, 40]. Moreover, a meta-analysis of 13 cross-sectional studies has also revealed that the healthy dietary pattern was significantly associated with 16% reduced odds of depression; while no statistically significant association was observed between the Western diet and depression in community-dwelling adults [36]. Unlike the use of AHEI, the healthy and Western dietary patterns were population-specific, as they were identified from existing eating habits in the study population. A priori scoring method like AHEI-2010, which was applied in the current study, may facilitate the use by clinicians in clinical settings to prevent anxiety or depression. It is worth noting that the AHEI-2010 was not developed specifically for psychological disorders prevention; therefore, healthy eating styles, as indicated by AHEI-2010, might protect against other chronic conditions as shown by previous studies [32, 41].

In the present study, we observed statistically significant association between smoking, psychological distress and healthy diet with mental disorders; however, other two lifestyle habits (physical activity and weight status) were not related to anxiety or depression. Lack of finding a significant association between these two factors with outcomes might be explained by the use of their binary variable in our study. In addition, controlling for other lifestyle variables might be over-adjustment in this regard because most lifestyle-related factors are inter-related. In addition, a comprehensive analysis of healthy lifestyle may capture influence of individual factors better than analyses based on a single factor, given the complexity and multiple dimensions of habitual health behaviors. Further investigation is needed to determine whether critical thresholds exist for each lifestyle factor.

Diet, smoking and other components of healthy lifestyle score may influence mental health through a number of different pathways, including modification of neurotrophins critical to psychologic disorders and oxidative and nitrosative stress pathways [42]. In addition, depressed and anxious individuals have higher levels of C reactive protein (CRP) levels [43], a marker for systemic inflammation, which is thought to play a role in the genesis of depression [44]. Elevated CRP has also been associated with lifestyle factors such as obesity [45], unhealthy diet [46], cigarette smoking [47], and physical inactivity [48]. These findings together suggest that participants with depression and anxiety symptoms have higher CRP levels due to unhealthy lifestyle behaviors; although no information about CRP was available in our study. Further investigation is warranted to clarify these aspects of the relationship between mental disorders and lifestyle factors.

Strengths of our study include the large sample size, careful assessment of confounding and dietary intakes, psychological distress and physical activity with validated questionnaires. Unlike several prior studies that mostly focused on a single lifestyle behavior, we assessed the combined lifestyle score in relation to mental disorders. However, several limitations need to be considered when interpreting our findings. Due to the cross-sectional design of the study, causality cannot be inferred. Those suffering from mild anxiety or depression might be less likely to eat well and exercise, and more likely to self-medicate with smoking; these unhealthy choices potentially can exacerbate existing disorder symptoms; therefore, anxiety or depression might be the cause, rather than the consequence, of one or more unhealthy lifestyle factors. Future investigations are needed to have better understanding of the direction of this association. Some measurement error is inevitable, particularly in the assessment of diet and physical activity. However, such errors would attenuate the true associations. One might probably reach a lower risk of mental disorders in case of using more restrictive criteria for the low risk group (for example, healthy diet score in the highest fifth and physical activity ≥3 h/w). However, our results indicated that even modest differences in lifestyle can have a substantial impact on reducing anxiety and depression. We tried to control for known confounding variables associated with the outcome; however, residual confounding in our study, as in all epidemiological studies, is inevitable. Furthermore, equal weight was assigned to each component of lifestyle to achieve the main purpose of the study. This might be kept in mind in the interpretation of our findings. Moreover, since the number of lean participants was low (n = 114), we included them in the category of normal-weight subjects. The prevalence of depression and anxiety in lean individuals was higher than normal-weight individuals (for depression: 41.2 vs. 29.9% and for anxiety: 16.7 vs. 14.0%). Although some sort of bias might have been resulted from this inclusion, it would be negligible and such error would move the ORs toward non-significant findings. Finally, the study population consisted of a medical university non-academic staff, including crews, employees and managers. Although the socio-economic status of the study population was representative of general Iranian population, extrapolating the findings to other populations should be made cautiously.

In conclusion, we found evidence indicating that healthy lifestyle score was associated with lower odds of anxiety and depression. In addition, healthy diet, psychological distress, and smoking status were independent predictors of mental disorders. Given the cross-sectional design of the present study, future prospective studies are required to confirm these findings.

## Abbreviations

FFQ: food frequency questionnaire
GPPAQ: General Practice Physical Activity Questionnaire
GHQ: General Health Questionnaire
HADS: Hospital Anxiety and Depression Scale
OR: odds ratios
95% CI: 95% Confidence Interval
U.S.: United States
NHANES: National Health and Nutrition Examination Survey
SEPAHAN: Study on the Epidemiology of Psychological-Alimentary Health and Nutrition
IUMS: Isfahan University of Medical Sciences
AHEI: Alternative Healthy Eating Index
CRP: C-reactive protein
